# Diagnosis of sarcopenic obesity in Japan: Consensus statement of the Japanese Working Group on Sarcopenic Obesity

**DOI:** 10.1111/ggi.14978

**Published:** 2024-09-10

**Authors:** Kojiro Ishii, Wataru Ogawa, Yutaka Kimura, Toru Kusakabe, Ryo Miyazaki, Kiyoshi Sanada, Noriko Satoh‐Asahara, Yuki Someya, Yoshifumi Tamura, Kohjiro Ueki, Hidetaka Wakabayashi, Yuya Watanabe, Minoru Yamada, Hidenori Arai

**Affiliations:** ^1^ Faculty of Health and Sports Science Doshisha University Kyotanabe Japan; ^2^ Division of Diabetes and Endocrinology, Department of Internal Medicine Kobe University Graduate School of Medicine Kobe Japan; ^3^ Health Science Center Kansai Medical University Hirakata Japan; ^4^ Department of Endocrinology, Metabolism, and Hypertension Research Clinical Research Institute, National Hospital Organization Kyoto Medical Center Kyoto Japan; ^5^ Faculty of Human Sciences Shimane University Matsue Japan; ^6^ Faculty of Sport and Health Science Ritsumeikan University Kusatsu Japan; ^7^ Sportology Center Juntendo University Graduate School of Medicine Tokyo Japan; ^8^ Faculty of Health and Sports Science Juntendo University Chiba Japan; ^9^ Department of Metabolism and Endocrinology Juntendo University Graduate School of Medicine Tokyo Japan; ^10^ Diabetes Research Center, National Center for Global Health and Medicine Tokyo Japan; ^11^ Department of Rehabilitation Medicine Tokyo Women's Medical University Hospital Tokyo Japan; ^12^ Faculty of Sport Study Biwako Seikei Sport College Otsu Japan; ^13^ Faculty of Human Sciences University of Tsukuba Tsukuba Japan; ^14^ National Center for Geriatrics and Gerontology Obu Japan

**Keywords:** algorithm, definition, diagnostic criteria, sarcopenic obesity

## Abstract

Sarcopenic obesity is the co‐existence of obesity and sarcopenia in individuals aged 40–75 years. The Japanese Working Group on Sarcopenic Obesity has developed diagnostic criteria tailored for the Japanese population, considering their unique characteristics compared with European populations. Our algorithm consists of two steps: screening and diagnosis. The screening of obesity mandates using waist circumference and/or body mass index (BMI) based on national standards, while screening for sarcopenia involves the “finger ring test” in addition to the Asian Working Group for Sarcopenia 2019 criteria. The final diagnosis of sarcopenia involves handgrip strength for low muscle strength, the five‐times chair stand test for low physical function, and limb skeletal muscle mass (corrected for BMI) for low muscle mass. Obesity is assessed by visceral fat area or body fat percentage. Sarcopenic obesity is then categorized into Stage I, with muscle weakness/loss of function, loss of muscle mass, and obesity; or Stage II, which includes complications. Further clinical validation is needed to refine the consensus and age range. **Geriatr Gerontol Int 2024; 24: 997–1000**.

## Graphical abstract

Screening: (A) Surrogate parameters for sarcopenia. (B) High waist circumference or body mass index. To proceed to diagnosis, (A) is recommended and (B) is required. Diagnosis: (C) Altered skeletal muscle functional parameters and decreased muscle mass. (D) Increased visceral fat area or body fat percentage. (C) and (D) must be present to assess the presence of sarcopenic obesity.

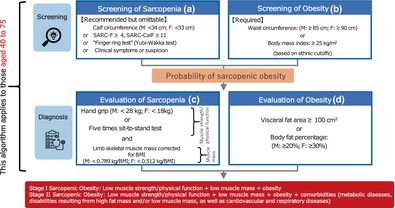

## Introduction

Sarcopenic obesity, wherein individuals with obesity also have sarcopenia, has attracted much attention. For many years, there has been no consensus on its definition and diagnostic criteria, with inconsistent criteria being used in studies.^1^

**Figure 1:**
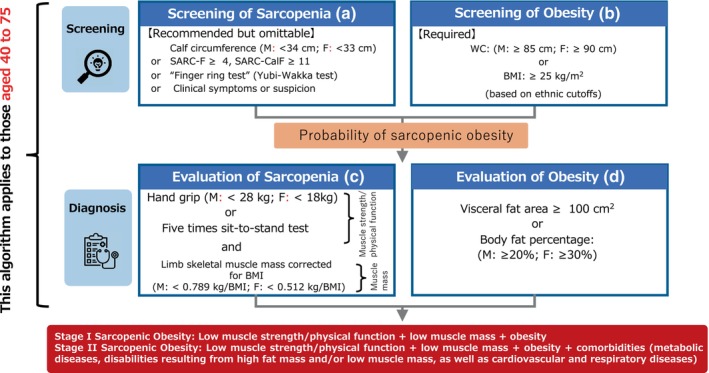
Diagnostic algorithm for sarcopenic obesity in Japan. Screening: (a) Surrogate parameters for sarcopenia (clinical symptoms, clinical suspicion, or questionnaires). (b) High waist circumference (WC) or body mass index (BMI) (based on ethnic cutoff points). To proceed to diagnosis, (a) is recommended and (b) is required. Diagnosis: (c) Altered skeletal muscle functional parameters (handgrip strength, chair stand test), and decreased muscle mass (limb skeletal muscle mass corrected for BMI). (d) Altered visceral or body fat: increased visceral fat area or body fat percentage. (c) and (d) must be present to assess the presence of sarcopenic obesity. Calf circumference (M, <34 cm; F, <33 cm) is based on the study by Kawakami *et al*.^2^ and “Clinical Symptoms or Suspicion” is from the ESPEN and EASO criteria.^3^ WC (M, ≥85 cm; F, ≥90 cm) or BMI (≥25 kg/m^2^) is based on the JASSO criteria.^4^ M, males; F, females.

In October 2017, the Japan Society for the Study of Obesity (JASSO) and the Japanese Association on Sarcopenia and Frailty (JASF) agreed to establish a joint working group on sarcopenic obesity (the Japanese Working Group on Sarcopenic Obesity [JWGSO]). This group consisted of members from both societies and started its activities 1 year later. After approximately 5 years of discussions, the definition and diagnostic criteria for sarcopenic obesity were presented at the congresses of both societies in November 2023 (JASF: November 5; JASSO: November 26) (Fig. 1) (Clinical Symptoms or Suspicion of the European Society for Clinical Nutrition and Metabolism [ESPEN] and European Association for the Study of Obesity [EASO] criteria) (Table 1).

**Table 1:** Clinical Symptoms or Suspicion Factors for the screening of sarcopenic obesity (European Society for Clinical Nutrition and Metabolism [ESPEN] and European Association for the Study of Obesity [EASO] criteria^3^)

• Age >70 years
• Chronic disease diagnosis (e.g. inflammatory diseases and organ failure or chronic disease) including but not limited to:
‐ Chronic heart failure
‐ Chronic kidney disease (particularly renal replacement therapy)
‐ Chronic bowel failure or dysfunction
‐ Chronic liver disease (particularly NASH and liver cirrhosis)
‐ Chronic respiratory disease
‐ Chronic neurologic and neurodegenerative diseases
‐ Chronic cognitive impairment
‐ Depression
‐ Organ transplantation
‐ Endocrine diseases (e.g., metabolic syndrome, diabetes mellitus, hypercortisolism, hypogonadism, and corticoid treatment)
‐ Osteoarthritis
‐ Cancer (especially but not limited to chemotherapy of breast or prostate cancer)
•Recent acute disease/nutritional events:
‐ Recent hospitalization (particularly but not limited to COVID‐19, intensive care unit stay, surgery)
‐ Recent major surgery or trauma with/without complications
‐ Recent sustained immobilization or reduced mobility (e.g. trauma, fracture, orthopedic disease)
‐ Recent history of reduced food intake (e.g., <50% for >2 weeks)
‐ Recent weight loss (including diet‐induced voluntary weight loss and weight cycling syndrome)
‐ Recent rapid increase of weight
‐ Long‐standing restrictive diets and bariatric surgery
•History of complaint of:
‐ Repeated falls
‐ Weakness, exhaustion
‐ Fatigability
‐ Perceived progressive movement limitations

Abbreviation: NASH (MASH, metabolic dysfunction‐associated steatohepatitis).

A consensus statement of the ESPEN and the EASO was published in 2022.^3^ However, Asian populations, including those from Japan, typically have lower skeletal muscle mass and strength because their ethnicity, body size, lifestyle, and cultural background differ from those of European populations. In addition, Asian populations are more likely to develop insulin resistance as well as metabolic and atherosclerotic diseases even with mild obesity.^5^ Therefore, our working group aimed to develop diagnostic criteria for sarcopenic obesity in the Japanese population that could be applied to Asian populations.

## Definition of sarcopenic obesity

Sarcopenic obesity is defined as the co‐existence of obesity and sarcopenia. However, we do not consider sarcopenic obesity to be sufficiently identified by combining existing obesity and sarcopenia diagnostic criteria.

## Screening

Many young Japanese women have low skeletal muscle mass and a consequently high body fat percentage, although they are not overweight or obese.^6^ Because the diagnosis of sarcopenic obesity based on body fat percentage alone would include such individuals, we decided to make the assessment of obesity with waist circumference (WC) and/or body mass index (BMI) mandatory. Considering the applicability to other countries, we decided to use national standards for the cutoff values of WC and BMI (for Japan, the JASSO^4^ criteria were used). However, national standards for the cutoff values of WC and BMI may need to be more detailed because of the large number of multi‐ethnic nations in Asia. Additionally, there are conflicting reports on the risk of diseases associated with obesity in patients aged ≥75 years, and JASSO^4^ has not yet established clear treatment guidelines for obesity‐related diseases in this age group owing to a lack of evidence. The JWGSO recognizes that sarcopenic obesity is an age‐related disease, and the applicable age for the definition and diagnostic criteria of sarcopenic obesity is between 40 and 75 years.

To screen for sarcopenia in sarcopenic obesity, we added the simple “finger ring test” (Yubi‐Wakka test^7^) developed in Japan to the Asian Working Group for Sarcopenia 2019^8^ screening items. In addition, calf circumferences of <34 cm for males and <33 cm for females are the cutoff values for low skeletal muscle mass in Japanese individuals, based on Kawakami *et al*.^2^ However, as these screening items for sarcopenia are not widely used in Japan, our consensus is that they are “recommended” and can be omitted.

## Diagnosis

The final assessment for the diagnosis of sarcopenia and obesity uses handgrip strength as a criterion for low muscle strength and the five‐times chair stand test as a criterion for low physical function.^8^ According to the ESPEN and EASO consensus,^3^ low muscle mass can be assessed either by appendicular lean mass adjusted for body weight (ALM/W) using dual‐energy X‐ray absorptiometry (DXA) or by bioelectrical impedance analysis (BIA) to measure skeletal muscle mass adjusted for body weight. Based on the results of our study in older Japanese patients,^9^ we adopted limb skeletal muscle mass corrected for BMI.^10^ In other words, BMI‐corrected limb skeletal muscle mass is suitable for identifying overweight Japanese older adults with sarcopenia. The limb skeletal muscle mass corrected for BMI as used by the Foundation for the National Institutes of Health (FNIH) is ALM/W measured by DXA.^10^ However, because BIA is widely used in Asia, the FNIH criteria will be used for BIA on a trial basis. Obesity will be assessed by visceral fat area (≥100 cm^2^) or body fat percentage (males: ≥20%; females: ≥30%).^11^ In addition to WC and/or BMI at the screening stage, obesity can be determined by visceral fat area or body fat percentage, thus preventing those who are underweight but have low skeletal muscle mass, common among young Japanese women, from being judged as obese. We defined Stage I sarcopenic obesity as a combination of muscle weakness and/or loss of physical function, loss of skeletal muscle mass, and obesity, and Stage II sarcopenic obesity as a combination of these conditions with complications.

## Remaining issues

Our consensus has not yet been clinically validated. As clinical data accumulate, our consensus and its applicable age range will continue to be updated (Appendix A).

## Disclosure statement

The authors declare no conflict of interest.

## Ethics statement

Ethics review by the authors' institutions was not required as this is a consensus statement developed by a working group without involving human subjects.

## Data Availability

Research data are not shared.

## APPENDIX A Appendices

### A.1 Members of the Joint Working Group

Chair: Ishii, Kojiro: Doshisha University; Director, JASSO and JASF.

Vice‐chair: Arai, Hidemori: National Center for Geriatrics and Gerontology; President, JASF.

### A.2 Members (in alphabetical order)

Kimura, Yutaka: Kansai Medical University; Councilor, JASSO.

Kusakabe, Toru: National Hospital Organization Kyoto Medical Center; Councilor, JASSO and JASF.

Miyazaki, Ryo: Shimane University; Councilor, JASSO and JASF.

Ogawa, Wataru: Kobe University; Director, JASSO.

Sanada, Kiyoshi: Ritsumeikan University; Director, JASF.

Satoh‐Asahara, Noriko: National Hospital Organization Kyoto Medical Center; Councilor, JASSO.

Someya, Yuki: Juntendo University; Councilor, JASSO.

Tamura, Yoshifumi: Juntendo University.

Ueki, Kohjiro: National Center for Global Health and Medicine; Director, JASF and Councilor, JASSO.

Wakabayashi, Hidetaka: Tokyo Women's Medical University Hospital; Director, JASF.

Watanabe, Yuya: Biwako Seikei Sport College; Councilor, JASSO and JASF.

Yamada, Minoru: University of Tsukuba; Director, JASF.
